# Parathyroid Cell Proliferation in Secondary Hyperparathyroidism of Chronic Kidney Disease

**DOI:** 10.3390/ijms21124332

**Published:** 2020-06-18

**Authors:** Tally Naveh-Many, Oded Volovelsky

**Affiliations:** 1 Minerva Center for Calcium and Bone Metabolism, Nephrology Services, Hadassah Hebrew University Medical Center, Jerusalem 91120, Israel; tally@cc.huji.ac.il; 2 The Wohl Institute for Translational Medicine, Hadassah Hebrew University Medical Center, Jerusalem 91120, Israel; 3 Pediatric Nephrology Unit and Research Lab, Hadassah Hebrew University Medical Center, Jerusalem 91120, Israel

**Keywords:** calcium-sensing receptor (CaSR), fibroblast growth factor (FGF23), mammalian target of rapamycin (mTOR), vitamin D receptor (VDR), parathyroid hormone (PTH), uremia

## Abstract

Secondary hyperparathyroidism (SHP) is a common complication of chronic kidney disease (CKD) that correlates with morbidity and mortality in uremic patients. It is characterized by high serum parathyroid hormone (PTH) levels and impaired bone and mineral metabolism. The main mechanisms underlying SHP are increased PTH biosynthesis and secretion as well as increased glandular mass. The mechanisms leading to parathyroid cell proliferation in SHP are not fully understood. Reduced expressions of the receptors for calcium and vitamin D contribute to the disinhibition of parathyroid cell proliferation. Activation of transforming growth factor-α-epidermal growth factor receptor (TGF-α-EGFR), nuclear factor kappa B (NF-kB), and cyclooxygenase 2- prostaglandin E2 (Cox2-PGE2) signaling all correlate with parathyroid cell proliferation, underlining their roles in the development of SHP. In addition, the mammalian target of rapamycin (mTOR) pathway is activated in parathyroid glands of experimental SHP rats. Inhibition of mTOR by rapamycin prevents and corrects the increased parathyroid cell proliferation of SHP. Mice with parathyroid-specific deletion of all miRNAs have a muted increase in serum PTH and fail to increase parathyroid cell proliferation when challenged by CKD, suggesting that miRNA is also necessary for the development of SHP. This review summarizes the current knowledge on the mechanisms of parathyroid cell proliferation in SHP.

## 1. Introduction

Calcium is the major regulator of parathyroid function. The parathyroid calcium-sensing receptor (CaSR), a 7-transmembrane G-protein-coupled receptor located on the parathyroid cell membrane, senses small changes in serum calcium [1]. A decrease in serum calcium stimulates parathyroid hormone (PTH) secretion and, when prolonged, parathyroid cell proliferation [2,3,4]. PTH synthesis, secretion, and proliferation are also controlled by serum phosphate, vitamin D, and the bone-derived phosphaturic hormone fibroblast growth factor 23 (FGF23) [3,5,6,7,8,9,10]. Phosphate induces a concentration-dependent stimulation of PTH from bovine and rat parathyroid tissue [10,11,12]. In addition, a high phosphorus diet or phosphate loading increase serum PTH levels in healthy and in uremic experimental models [13,14]. Although the effect of phosphate to increase PTH synthesis and secretion is well established, the mechanism of this effect has been under debate. Recent studies have suggested that the CaSR may act as a phosphate sensor in the parathyroid. Phosphate inhibits the CaSR in a non-competitive manner and thus increases PTH secretion [15]. PTH is a major stimulator of vitamin D activation to 1,25-dihydroxyvitamin D (1,25D) in the kidney. 1,25D, in turn, exerts negative feedback to downregulate the expression of PTH. 1,25D binds to the vitamin D receptor that forms a heterodimer with retinoic X receptor on vitamin D response elements in the *PTH* gene promoter [16,17,18]. Phosphate, 1,25D, and PTH increase FGF23 expression in bone osteocytes. FGF23 acts on the parathyroid fibroblast growth factor receptor (FGFR1)–klotho receptor complex to decrease PTH expression [7,19].

The parathyroid glands develop from a shared initial organ primordium together with the thymus. Both organs arise from the third pharyngeal pouch endoderm and surrounding neural crest cells. Rodents have two and humans four parathyroid glands. In humans, two additional superior parathyroid glands develop from the fourth pouch [20]. In both humans and rodents, chief cells produce PTH. In addition to the PTH-producing chief cells, human parathyroids contain oxyphil cells that are larger and lighter stained than the chief cells. Oxyphil cells are absent in parathyroids of mice, rats, chickens, and other species. The significance of oxyphil cells in the pathophysiology of the parathyroid glands is not clear [21,22].

Secondary hyperparathyroidism (SHP) is a common complication of chronic kidney disease (CKD). It is characterized by increased PTH production and secretion as well as increased glandular size, resulting in impaired bone and mineral metabolism. CKD affects approximately 10% of the worldwide population and is associated with a high rate of morbidity and premature death. Over 2 million CKD patients worldwide receive dialysis or a kidney transplant to survive. Millions more die due to lack of access to affordable treatment or dialysis [23]. The etiology of CKD is diverse, with diabetes, obesity, and hypertension being the main causes in adults [24]. Kidney failure impairs growth in children and increases the risk of fracture and skeletal deformities. Cardiovascular disease with decreased vascular compliance and left ventricular hypertrophy are major causes of morbidity in the CKD population with tight and independent correlation with SHP [25,26]. CKD impairs mineral homeostasis, including abnormalities in calcium, phosphate, PTH, FGF23, and vitamin D [9,27,28]. Alterations in calcium and phosphate homeostasis occur early in the course of CKD and progress as kidney function decreases. CKD interrupts mineral homeostasis directly by phosphate retention secondary to reduced glomerular filtration and decreased renal 1-α hydroxylation of vitamin D in the failed kidney, leading to low levels of 1,25D. FGF23 levels rise at the early stages of CKD, leading to a further reduction in 1,25D. The increased PTH production and parathyroid gland hyperplasia of CKD-induced SHP contribute to bone and cardiovascular disease [29,30]. The pathogenesis of SHP of CKD is multifactorial. Phosphate retention, hyperphosphatemia, hypocalcemia, and low levels of 1,25D all contribute to the development of uremic SHP. While phosphate retention and low serum calcium can explain the rise in PTH, in many cases, PTH levels increase even before the changes in minerals. The early rise in PTH can be explained by a loss of disinhibition of PTH by the low 1,25D levels. In addition, parathyroid hyperplasia leads to reduced expression of the vitamin D and calcium receptors in parathyroid cells, creating resistance of the gland to physiological regulation in CKD [31].

**Figure 1:**
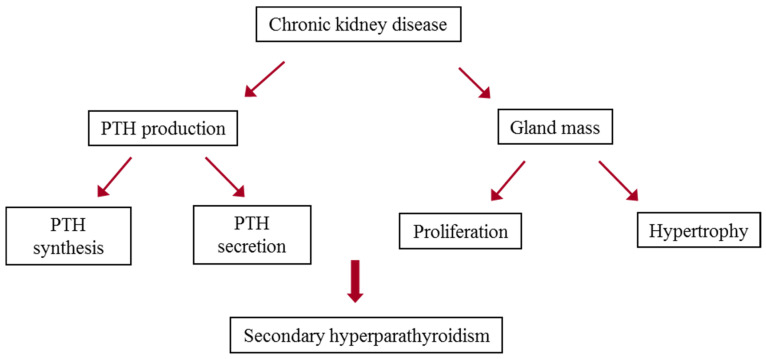
Pathogenesis of secondary hyperparathyroidism (SHP) in chronic kidney disease. There are two major mechanisms which determine the magnitude of SHP: Increased parathyroid hormone (PTH) production composed of PTH synthesis and secretion per cell, and increased parathyroid gland mass, mostly due to parathyroid cell proliferation and, to a lesser degree, increased cell size (hypertrophy).

The magnitude of SHP is determined by two major mechanisms, an increase in PTH synthesis and secretion per cell and an increase in parathyroid gland mass (Figure 1) [32]. Parathyroid cells have a limited number of secretory granules containing preformed hormone compared to other endocrine cells. Therefore, increases in *PTH* gene expression are essential for a continued secretion of PTH. The increase in *PTH* gene expression in SHP is regulated by posttranscriptional mechanisms that alter PTH mRNA stability and levels. PTH mRNA stability is mediated by PTH mRNA–protein binding orchestrated by the cis-trans isomerase Pin1 [33,34]. microRNAs (miRNAs) also contribute to the stimulation of the parathyroid in SHP [35]. The increase in *PTH* gene expression is tightly linked to increased parathyroid glandular mass induced by parathyroid cell proliferation and, to a lesser degree, by an increase in cell size. The molecular pathways mediating the increased parathyroid cell proliferation are still obscure. This review will discuss the pathogenesis of parathyroid cell proliferation in CKD-induced SHP.

## 2. Parathyroid Cell Proliferation in Secondary Hyperparathyroidism

**Figure 2:**
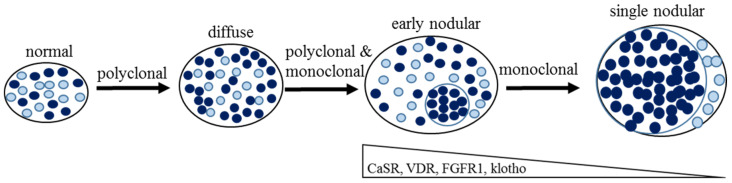
Parathyroid hyperplasia in chronic kidney disease (CKD) patients with SHP. Schematic presentation of the different stages of SHP: In the monoclonal state, the parathyroid cells become gradually autonomous and transform into a nodular pattern. At the level of nodular hyperplasia, there is reduced expression of the vitamin D receptor (VDR), calcium-sensing receptor (CaSR), and the fibroblast growth factor 23 (FGF23) receptor complex (FGFR1-klotho) and the parathyroids become refractory to conventional medical therapy. Light and dark blue circles correspond to polyclonal growth within a parathyroid tissue, which develops into monoclonal (dark blue) benign cell proliferation in severe SHP.

The parathyroid cells are generally quiescent under physiological conditions, with low turnover and mitoses rates [36]. However, the cells retain their potential to proliferate in response to uremia, hypocalcemia, hyperphosphatemia, and vitamin D deficiency [37]. Increased serum PTH and parathyroid cell proliferation are hallmarks of SHP and among the earlier alterations of mineral metabolism in patients with CKD. At the early stages of SHP in CKD patients, the parathyroid glands show diffuse and polyclonal proliferation (Figure 2). Persistent uremia-related stimulation drives the progression of parathyroid cell proliferation from diffuse to nodular growth [38,39]. In the monoclonal state in advanced CKD, the parathyroid cells become gradually autonomously active with aggressive proliferation. At this stage, the enlarged parathyroid glands become irresponsive to conventional medical therapy. The main factors responsible for parathyroid cell proliferation appear to be similar to those leading to enhanced PTH biosynthesis and secretion, mainly high phosphate and hypocalcemia. In addition, 1,25D has an antiproliferative effect, and its deficiency in CKD contributes to parathyroid cell proliferation [40].

### Experimental Models of Chronic Kidney Disease Induced Secondary Hypeparathyroidism

Experimental SHP of CKD is induced in animal models by several methods: 5/6 nephrectomy is performed by removing one kidney and 2/3 of the contralateral kidney. SHP of 5/6 nephrectomized rats is characterized by an increase in serum and PTH mRNA levels and parathyroid cell proliferation that are further increased when the nephrectomized rats are fed a high-phosphorus diet. A low-phosphorus diet decreases both PTH expression and parathyroid cell proliferation, emphasizing the role of a normal phosphate in the prevention of parathyroid cell proliferation [13,14,41]. Rather than mimicking a renal disease itself, 5/6 nephrectomy leads to an acute reduction in functional renal mass. As a result, the progression to renal failure is dependent on the amount of tissue excised.

Another useful experimental model for SHP of CKD is obtained by feeding rats or mice an adenine high-phosphorus diet [42,43]. The physiologic serum and urine levels of adenine are low due to its salvage by adenine phosphoribosyltransferase (APRT) to adenosine monophosphate. When high levels of adenine are consumed, the enzyme is saturated and insoluble oxidized products of adenine accumulate in the kidney [44]. This leads to the formation of crystals in renal tubules with subsequent inflammation, fibrosis, and obstruction, resulting in kidney failure and SHP. The addition of high phosphorus to the adenine diet leads to even more prominent SHP [43,45]. The onset of uremia in the adenine high-phosphorus diet model is faster than 5/6 nephrectomy and seems to induce a more severe form of the disease. However, it is difficult to compare the two models because one is surgical and the other dietary. Adenine high-phosphorus diet-induced renal failure in rats occurs already at three days of the diet, as measured by high serum creatinine and urea levels. Serum phosphate was increased at two weeks and even more so at six weeks, with a decrease in 1,25D and serum calcium from three weeks of the diet. Parathyroid cell proliferation markers were high already at three days and persisted at two and six weeks of the adenine high-phosphorous diet. Serum PTH levels were increased from day 7 and were 4-fold increased at two weeks and 18-fold at six weeks of the diet. The increase in serum PTH was accompanied by an increase in PTH mRNA levels secondary to a posttranscriptional increase in PTH mRNA stability [45]. Serum FGF23 levels were also increased together with the increase in PTH [45,46].

## 3. Dysregulation of Parathyroid Cell Proliferation in Chronic Kidney Disease Induced Secondary Hyperparathyroidism

### 3.1. Resistance to Regulation by Phosphate, Calcium, 1,25D, and FGF23

**Figure 3:**
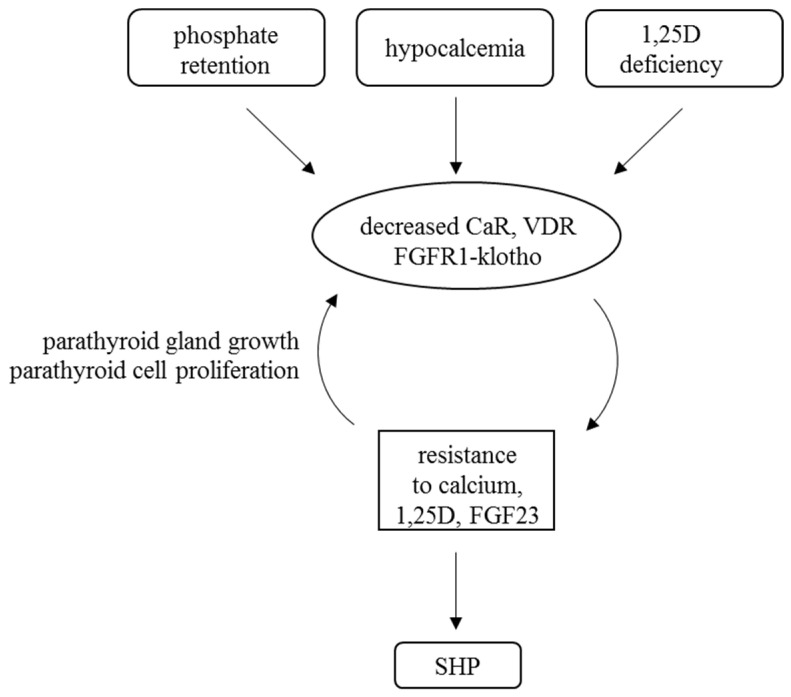
Receptor resistance in SHP: Phosphate retention, 1,25-dihydroxyvitamin D (1,25D) deficiency, and hypocalcemia are the major causes for SHP of CKD. Parathyroid cell proliferation results in decreased levels of the CaSR, VDR, and FGF23 receptor complex FGFR1-klotho. The decrease in receptors leads to resistance of the parathyroid to theses effectors, which further amplifies parathyroid cell proliferation. VDR, vitamin D receptor; CaSR, calcium sensing receptor; FGFR1, fibroblast growth factor 1 receptor; SHP, secondary hyperparathyroidism; and VDR, Vitamin D receptor.

Hypophosphatemia, hypercalcemia, as well as high serum levels of 1,25D and FGF23 suppress parathyroid activity. Therefore, the parathyroid CaSR, VDR, and the FGF23 receptor complex FGFR1-klotho have a central role in the regulation of PTH expression in physiological and disease states. Parathyroid gland hyperplasia of CKD-induced SHP is associated with downregulation of the parathyroid CaSR, VDR, FGFR1, and klotho expressions (Figure 2 and Figure 3) [47,48,49].

The tight control of calcium homeostasis is interrupted in patients with either primary or secondary hyperparathyroidism caused by end-stage renal disease. This abnormal control of PTH secretion is attributed, at least in part, to the downregulation of the CaSR in hyperplastic parathyroid tissue. A substantial reduction in the expression of the CaSR mRNA and protein levels was demonstrated in hyperplastic parathyroid glands of uremic patients [48,50,51]. In experimental uremia induced by 5/6 nephrectomy, there was a decrease in CaSR expression in rats fed a high-phosphorus diet but not in uremic rats fed by a standard diet [52]. Similar findings were observed in parathyroid glands of dietary adenine high-phosphorus CKD models [46]. This process apparently occurs only after the development of parathyroid hyperplasia and is enhanced by high phosphate intake [52,53]. The chronic underactivation of the CaSR permits continuously elevated PTH secretion and parathyroid cell proliferation.

A reduction of VDR mRNA and protein levels was shown in glands from uremic patients with severe SHP [54,55]. Low VDR levels were also shown in experimental SHP [46,56]. The reduction in VDR expression inhibits the vitamin D-mediated signals that normally suppress PTH synthesis and release, and parathyroid cell proliferation. The greatest decrease in both CaSR and VDR expressions is in the hyperplastic nodules of the parathyroid gland, rendering them less responsive to circulating calcium and 1,25D (Figure 2 and Figure 3) [57]. In both experimental models and patients, the downregulation of CaSR and VDR expression were associated with parathyroid cell proliferation. Downregulation occurs in CKD only when the parathyroid glands are hyperplastic and probably contributes to the increased proliferation of the glands in SHP [40,46,53]. Phosphate restriction prevented both parathyroid cell proliferation and the decreased CaSR expression in CKD [10]. These and other studies suggested that parathyroid gland hyperplasia reduces the expressions of CaSR and VDR, thereby further enhancing the elevation of PTH secretion and parathyroid cell proliferation (Figure 3).

The classical treatment for SHP includes active vitamin D compounds and phosphate binders to limit gastrointestinal phosphate absorption [40,58]. Vitamin D-supplemented diet in SHP rat models increased the expression of the VDR and CaSR and therefor reduced parathyroid cell proliferation [59,60]. However, while vitamin D compounds suppress PTH secretion, they also promote calcium and phosphate intestinal absorption. Calcimimetic compounds are positive allosteric modulators of the CaSR that increase the sensitivity of the CaSR, thereby decreasing PTH secretion from the parathyroid glands. Calcimimetics are widely used to lower PTH levels in CKD patients with SHP. Studies using experimental CKD rat models have demonstrated that calcimimetics reduce parathyroid cell proliferation as measured by gland weight, DNA content, and staining for proliferation markers [61]. Reduction in parathyroid gland volume has also been demonstrated using imaging studies in dialysis patients receiving calcimimetics [62,63]. Calcimimetics increase cell surface CaSR expression in the parathyroid cells in uremic rats. They also correct the reduced vitamin D receptor expression of SHP [64].

### 3.2. Phosphate Sensing by the CaSR

Recent studies have identified the CaSR as a phosphate sensor in the parathyroid gland. The crystallized extracellular domain of the CaSR revealed four putative multivalent anion-binding sites occupied by phosphate (Pi) and sulfate (SO_4_^2−^) [65]. Anion binding to sites 1 and 3 preferentially stabilized the inactive conformation of the CaSR. Specifically, increased extracellular phosphate at concentrations observed in CKD inhibited the CaSR in a noncompetitive manner and, thus, increased PTH secretion. These findings provide a molecular mechanism for the stimulatory action of high phosphate levels on PTH secretion. In CKD, where serum phosphate levels are increased due to impaired renal excretion, phosphate-mediated stabilization of the CaSR would maintain it in an inactive conformation. This would stimulate high PTH secretion and parathyroid cell proliferation, leading to SHP and the resulting further bone loss and hyperphosphatemia [15].

### 3.3. Parathyroid Resistance to FGF23

FGF23 decreases PTH levels both in vitro in bovine parathyroid cells and in vivo in rats with normal renal function [7,19]. On the other hand, mice with conditional knockout of the FGF receptor or klotho had a muted increase in serum PTH and in parathyroid cell proliferation in early CKD compared to control CKD mice, suggesting that FGF23 is in fact a long-term inducer of parathyroid cell proliferation and PTH secretion [66]. Indeed, CKD patients and experimental CKD rats have high serum levels of both FGF23 and PTH levels. This apparent parathyroid resistance to FGF23 is secondary to a decreased expression of the FGF23 receptor complex. FGFR1 and klotho mRNA and protein levels are decreased in parathyroid tissue of chronic hemodialysis patients, particularly in nodular areas of the hyperplastic glands (Figure 2 and Figure 3). The reduced levels of the FGF23 receptor complex correlated inversely with parathyroid cell proliferation, as marked by increased Ki67 levels [62]. Rats with chronic renal failure had low klotho and FGFR1 mRNA and protein levels in the parathyroid [46,62]. Moreover, unlike control rats, recombinant FGF23 failed to decrease serum PTH or to activate MAPK (mitogen-activated protein kinase) signaling in the parathyroid glands of uremic rats in vivo. Moreover, in parathyroid glands of uremic rats in organ culture, FGF23 did not inhibit PTH mRNA expression and PTH secretion in contrast to the expected inhibitory response in normal rat parathyroids [46,67]. Therefore, parathyroid FGFR1 and klotho expression is decreased in experimental SHP and in end-stage renal disease patients, and this may lead to resistance of the parathyroids to FGF23 in SHP.

## 4. Post-Receptor Mechanisms of Parathyroid Cell Proliferation in Secondary Hyperparathyroidism

### 4.1. Cell-Cycle Regulation

Specific genetic abnormalities were proposed as the mechanisms regulating parathyroid proliferation in primary hyperparathyroidism. Two particular genes have been implicated in the pathogenesis of parathyroid tumorigenesis: the *cyclin D1/PRAD1* (parathyroid adenomatosis 1) oncogene and the *MEN1* (multiple endocrine neoplasia type 1) tumor-suppressor gene [68,69]. Menin, the product of the *MEN1* gene, is a tumor suppressor protein in a variety of cancer types. Inactivating *MEN1* mutations lead to the development of parathyroid neoplasia in almost all patients with MEN1. 10–25% of cases of MEN type 2A with the RET (rearranged during transfection) oncogene mutation have parathyroid neoplasia [70]. PRAD1, later identified as cyclin D1, plays a vital role in controlling the cell cycle. Rearrangements involving the *PTH* gene locus in 20–40% parathyroid adenoma patients identified cyclin D1 overexpression, suggesting that overexpression of PRAD1/cyclin D1 is one of the genetic abnormalities responsible for tumorigenesis in sporadic primary parathyroid adenomas contributing to parathyroid hyperplasia in humans [71,72]. Transgenic mice with parathyroid-targeted overexpression of cyclin D1, modeling the gene rearrangement found in human tumors, showed that a primary defect in this cell-cycle regulator caused primary hyperparathyroidism, as in human patients [73,74,75]. These findings suggest that overexpression of the cyclin D1 oncogene drives excessive parathyroid cell proliferation.

Parathyroid carcinoma in CKD-induced SHP patients is a rare event. It is not clear if benign parathyroid tumors may develop towards malignant forms in SHP [75,76]. Furthermore, the role of mutations in *cyclin D1 and MEN1* in SHP is not entirely understood. Expression analysis of human hyperplastic parathyroid glands from patients with advanced SHP showed only a minor role of PRAD1/cyclin D1 induced by *PTH* gene rearrangement. The levels of *cyclin D1* and *retinoblastoma* gene products increase in CKD-induced hyperplastic parathyroids [75]. The increase in cell-cycle progression in SHP correlates with a decrease in the expression of the cyclin-dependent kinase inhibitors (CKIs), p21 and p27. Loss of expression or function of p21 and p27 is implicated in many human malignancies [77]. The decrease in parathyroid p21 and p27 levels in SHP is most evident in cells with nodular hyperplasia. Low dietary phosphate reduced parathyroid cell proliferation in SHP rats, together with the induction of p21 and reduced expression of transforming growth factor (TGF)-α expression. A high phosphate intake, with subsequent stimulation of parathyroid cell proliferation in 5/6 nephrectomized rats, increased TGF-α levels [78]; 1,25D, that inhibits parathyroid cell proliferation in uremic rats, enhanced parathyroid p21 expression and prevented the high phosphate-induced increase in parathyroid TGF-α content [79,80]. A high calcium diet as well as vitamin D supplementation induced P21 and reduced TGF-α expression in parathyroid glands of uremic rats. In addition, 1,25D downregulated EGFR growth signaling by modifying receptor intracellular trafficking [81].

### 4.2. TGF-α—EGFR

**Figure 4:**
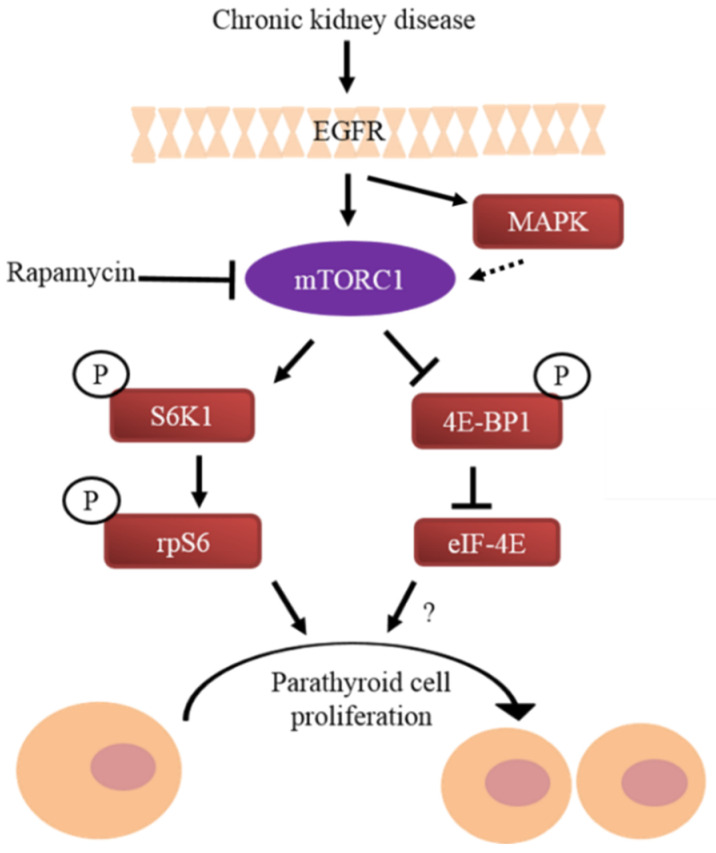
Mammalian target of rapamycin (mTORC1) induces parathyroid cell proliferation in secondary hyperparathyroidism (SHP) through ribosomal protein S6 (rpS6) phosphorylation. The mTORC1 pathway is activated in the parathyroids of rats and mice with CKD-induced SHP. Activated mTORC1 phosphorylates S6 kinase 1 (S6K1) that then phosphorylates rpS6 to induce parathyroid cell proliferation. mTORC1 also disinhibits eukaryotic translation initiation factor-4E (eIF-4E). Rapamycin decreases parathyroid cell proliferation in SHP by inhibiting mTORC1 and by reducing rpS6 activation. High levels of parathyroid epidermal growth factor receptor (EFGR) in SHP activates mTORC1 through mitogen-activated protein kinase (MAPK). The mTORC1 and EGFR pathways may act together to stimulate parathyroid cell proliferation in SHP. P designates protein phosphorylation, the effect of eIF-4E has not been determined (?), the dashed line indicates the suggested effect of MAPK on mTORC1 in the parathyroid cell. Adapted with permission from the *Journal of the American Society of Nephrology* [88].

The increased expression of TGF-α and its receptor EGFR in uremic rat and human hyperplastic and adenomatous parathyroid glands correlates with parathyroid cell proliferation [81,82]. EGFR signals through MAPK activation, which in turn induces cyclin D1 and drives the cell cycle from G1 to S [82,83,84]. EGFR activation by TGF-α also reduced VDR expression and plays an important role in parathyroid gland growth in uremic rats. Inhibition of parathyroid TGF-α/EGFR through the administration of potent and highly selective inhibitors of ligand-induced EGFR activation prevented not only parathyroid cell proliferation but also the reduction in VDR in parathyroid cells. A parathyroid-specific dominant-negative EGFR prevented the increase in serum PTH and parathyroid gland size and the decreased VDR expression observed in wild-type uremic mice [85]. EGFR also activates the protein kinase B (PKB, also known as AKT)-mTORC1 pathway (Figure 4) [86,87].

### 4.3. mTORC1

mTOR is part of the mammalian target of rapamycin complex 1 (mTORC1) that affects cell proliferation and metabolism by sensing the availability of growth factors and nutrients in the cell environment. AKT phosphorylation mediates signaling, which activates mTORC1 [89]. Rapamycin is a potent inhibitor of the mTORC1 complex and therefore inhibits cell proliferation [90]. The main downstream targets of mTORC1 include 4E binding protein 1 (4E-BP1) and ribosomal protein S6 (rps6). Rps6 is phosphorylated on five serine residues [91,92]. The substitution of these serine amino acids by alanine in rps6 knock-in mice impairs compensatory renal hypertrophy after unilateral nephrectomy and affects the metabolism and musculoskeletal system of the mice [93,94]. We have previously shown activation of the mTOR pathway in the parathyroids of rats and mice with SHP, which was induced by either chronic hypocalcemia or adenine high phosphorus-induced kidney failure. Increased mTOR activity was demonstrated by increased phosphorylation of rpS6. This activation correlated with increased parathyroid cell proliferation. Inhibition of mTORC1 by rapamycin both prevented and decreased parathyroid cell proliferation in SHP rats and in vitro in uremic rat parathyroid glands in organ culture. Moreover, knock-in rpS6p^−/−^ mice had impaired PTH secretion in experimental CKD and no increase in parathyroid cell proliferation compared with the expected increase in uremic wild-type mice [88]. These results highlight the essential role of mTORC1 activation by rpS6 phosphorylation in parathyroid cell proliferation and in the pathogenesis of SHP. The AKT-mTORC1 pathway is activated by EGFR. The mTORC1 and EGFR pathways may, therefore, act together to stimulate parathyroid cell proliferation in SHP (Figure 4).

### 4.4. NF-κB

Reduced circulating 1,25D and parathyroid VDR density play key roles in the progression of parathyroid cell proliferation. 1,25D partially restores parathyroid VDR expression and suppresses parathyroid cell proliferation by binding to its receptor [60]. In renal tubular cells, VDR binds to nuclear factor kappa B (NF-kB) dimers to suppress NF-κB-mediated gene transcription [95]. A reduction of VDR would lead to the activation of the NF-κB pathway. NF-κB is a ubiquitous transcription factor that plays a crucial role in immune and inflammatory responses. It consists of the DNA-binding subunit p50 and the transactivation subunit p65/RelA (p65) [96]. Different stimuli activate NF-kB by leading to degradation of its inhibitor, IkB, and the release and translocation of NF-kB to the nucleus [97]. Cyclin D1 is potently activated by p65. The tumor suppressor menin interacts with NF-kB and inhibits the NF-kB-mediated transcriptional activation [98]. NF-κB contributes to the pathogenesis of primary parathyroid hyperplasia [99]. In CKD-induced SHP, parathyroid glands from patients on dialysis showed activation of the NF-κB pathway in the nodular hyperplastic glands, with a significantly higher increase in diffuse hyperplastic glands [100]. In 5/6 nephrectomized rats fed a high-phosphorus diet, Proliferating cell nuclear antigen (PCNA) levels and activation of the NF-κB pathway were higher compared with the sham group, and VDR levels were decreased as expected. 1,25D decreased serum PTH and parathyroid cell proliferation together with reduced activation of the NF-κB pathway. A selective NF-κB inhibitor PDTC (ammonium pyrrolidinedithiocarbamate) decreased parathyroid NF-κB pathway activation, serum PTH, parathyroid cell proliferation, and the enlargement of the parathyroid glands in 5/6 nephrectomized rats [100]. These findings suggest that NF-κB contributes to parathyroid cell proliferation. Decreased 1,25D and VDR expression may affect parathyroid cell proliferation through the activation of the NF-κB pathway.

### 4.5. Cox-PGE2

Production of prostaglandins is catalyzed by the constitutive cyclooxygenase (COX) 1 and the tissue-specific COX2 enzyme, which is expressed mainly in inflammatory and proliferative cells [101]. Enhanced COX2 expression and its downstream metabolic product prostaglandin E2 (PGE2) biosynthesis were suggested to play a role in high phosphate-induced parathyroid cell proliferation. COX2 is expressed in parathyroid cells of normal, hyperplastic, and adenomatous parathyroid glands [102]. In parathyroids isolated from end-stage renal disease patients with advanced SHP, there was enhanced expression of both COX2 and PGE2 [103]. Increased COX2 expression was also reported in 5/6-nephrectomized rats fed a high-phosphorus diet, together with the increase in PTH levels, parathyroid size, and cell proliferation compared to controls. PGE2 increased PTH production in dispersed human and bovine parathyroid cells [104,105]. In primary cultures of parathyroids isolated from end-stage renal disease patients, high phosphate increased PTH secretion, parathyroid cell proliferation, and COX2 activity. Inhibitors of the PGE2 receptor 2 subtype (EP2) attenuated hyperparathyroidism induced by high phosphate. Accordingly, PGE2 or EP2 agonists directly stimulated hyperparathyroidism, suggesting that COX2 downstream PGE2 and its receptor EP2 play important roles in high phosphate-induced parathyroid cell proliferation [106].

### 4.6. The Role of MicroRNA in Secondary Hyperparathyroidism

MicroRNAs (miRNAs) are small noncoding RNAs which reduce the expression of various genes by binding to untranslated regions of mRNAs and enhancing their degradation and/or inhibiting their translation [107]. miRNAs are essential for normal development and are involved in fine-tuning of many biologic processes. Studies in parathyroid carcinomas identified dysregulated miRNAs when compared to normal parathyroid glands [108,109]. To study the role of miRNAs in SHP, miRNA profiling was performed by deep-sequencing in human parathyroid tissue of end-stage renal disease patients as well as experimental SHP models [110]. These studies showed that human and rodent parathyroids share similar miRNA profiles. High levels of miR-29, miR-21, miR-148, miR-30, and miR-141 and low levels of miR-10, miR-125, and miR-25 were observed in parathyroids of experimental models of SHP and patients. Inhibition of the abundant let-7 miRNA family increased PTH secretion in uremic rats as well as in mouse parathyroid organ cultures. Inhibition of the upregulated miR-148 family prevented the increase in serum PTH levels in uremic rats and decreased PTH secretion in parathyroid cultures. The evolutionary conservation of abundant miRNAs in normal parathyroid glands and the dysregulation of miRNAs in SHP support a key role for miRNAs in parathyroid function and in the development of hyperparathyroidism.

Cytoplasmic cleavage of the pre-miRNA precursor by the RNase III ribonuclease dicer is the final step of miRNA maturation. Mice with specific deletion of dicer and miRNAs in the parathyroid (PT-*Dice^−/−^* mice) develop normally and have normal serum PTH, calcium, and phosphate levels. However, when stressed by an adenine high-phosphorus diet to induce CKD, these mice had a muted increase in serum PTH and failed to increase PTH mRNA levels and parathyroid cell proliferation, suggesting that miRNA are essential for the development of SHP.

## 5. Summary

**Figure 5:**
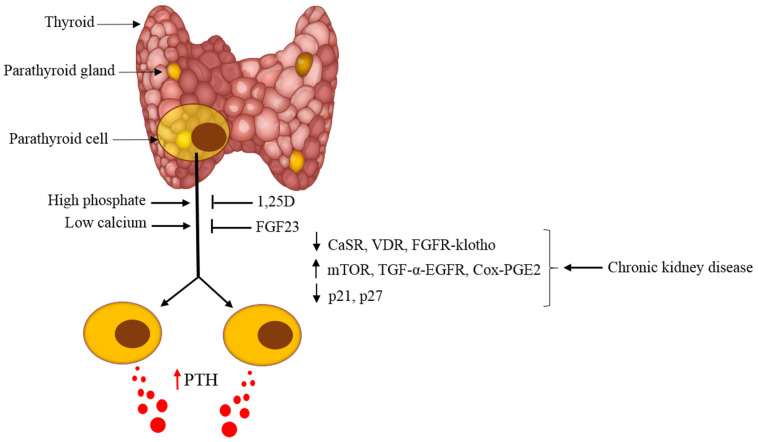
Regulators of parathyroid cell proliferation in SHP: Hyperphosphatemia and hypocalcemia stimulate and 1,25D and FGF23, inhibit PTH production and parathyroid cell proliferation. In CKD-induced SHP, decreased expression of the CaSR, VDR, and FGFR-klotho receptors together with activation of mTOR, TGF-α-EGFR, and cyclooxygenase 2- prostaglandin E2 (Cox-PGE2) signaling and changes in cell-cycle regulators lead to hyperplastic parathyroid glands with high serum PTH levels. miRNAs are essential for the development of SHP, but the identity of specific miRNAs remains to be determined (not shown). Manipulation of the above pathways may identify new targets to control SHP in CKD.

SHP is a common complication in CKD patients that correlates with morbidity and mortality. Increased PTH production and secretion per cell as well as a larger mass of the parathyroid gland, mainly secondary to increased parathyroid cell proliferation, lead to SHP in CKD. The physiologic regulation of parathyroid cell proliferation by calcium, phosphate, 1,25D and FGF23 is interrupted in advanced CKD due to receptor downregulation. Activation of proproliferative signaling pathways of mTOR, TGF-α-EGFR, NF-κB and Cox-PGE, together with interruption of cell division gatekeepers such as p21, lead to parathyroid cell proliferation in CKD. A better understanding of the mechanisms of CKD-induced SHP may identify new intervention opportunities for the control of the high serum PTH in SHP (Figure 5).

## Abbreviations

PTH	parathyroid hormone
CaSR	calcium sensing receptor
EGFR	epidermal growth factor receptor
mTOR	mammalian target of rapamycin
SHP	secondary hyperparathyroidism
CKD	chronic kidney disease
TGF-α	transforming growth factor α
1,25D	1,25 dihydroxyvitamin D
NF-kB	nuclear factor kappa B
Cox-PGE	cyclo-oxygenase- prostaglandin E
VDR	vitamin D receptor
FGFR1	fibroblast growth factor receptor
miRNA	micro-RNA
APRT	adenine phosphoribosyltransferase
MAPK	mitogen-activated protein kinase
eIF-4E	eukaryotic translation initiation factor-4E
PRAD1	parathyroid adenomatosis 1
MEN1	multiple endocrine neoplasia type 1
RET	rearranged during transfection
CKI	cyclin-dependent kinase inhibitor
AKT	protein kinase B
4E-BP1	4E binding protein 1
PCNA	proliferating cell nuclear antigen
PDTC	ammonium pyrrolidinedithiocarbamate
PGE2	prostaglandin E2
EP2	PGE2 receptor 2 subtype
PT-*Dicer-/-* mice	Parathyroid specific *dicer* knockout mice
